# Detection of *csgA* gene in carbapenem-resistant *Acinetobacter baumannii* strains and targeting with *Ocimum sanctum *biocompounds

**DOI:** 10.22038/IJBMS.2021.52852.11917

**Published:** 2021-05

**Authors:** Saishree R Anchana, Smiline A.S. Girija, Shoba Gunasekaran, Vijayashree J Priyadharsini

**Affiliations:** 1 Department of Microbiology, Saveetha Dental College and Hospitals, Saveetha Institute of Medical and Technical Sciences [SIMATS], Saveetha University, P.H.Road, Chennai, Tamilnadu - 600077, India; 2 Department of Microbiology, Saveetha Dental College and Hospitals, Saveetha Institute of Medical and Technical Sciences [SIMATS], Saveetha University, P.H.Road, Chennai, Tamilnadu - 600077, India; 3 Department of Biotechnology, DG Vaishnav College, Arumbakkam, Chennai – 600 106; 4 Department of Microbiology & Blue Lab, [Instead of BRULAC-DRC] Saveetha Dental College and Hospitals, Saveetha Institute of Medical and Technical Sciences [SIMATS], Saveetha University, P.H.Road, Chennai, Tamilnadu - 600077, India

**Keywords:** Acinetobacter baumannii, Benzofuran, Biofilms, Drug resistance, Eugenol, Ocimum sanctum

## Abstract

**Objective(s):**

Carbapenem-resistant *Acinetobacter baumannii* (CRAB) is considered highly virulent due to csgA gene-mediated biofilm formation. The present study aimed to target the same gene, employing the antibiofilm effect of *Ocimum sanctum* (*O. sanctum*) essential oil compounds among CRAB strains.

**Materials and Methods:**

A semi-quantitative adherent bioassay was performed to detect the biofilm formation in 73 CRAB strains. This was followed by molecular characterization, Polymerase Chain Reaction (PCR) amplification, and *csgA* gene sequencing. An antibiofilm assay under in vitro conditions, with essential oils of *O. sanctum* was performed. This was followed with further docking analysis of csgA protein with the selected compounds from the *O. sanctum *essential oils. A Molinspiration assessment was also done to elicit the drug likeliness of the biocompounds.

**Results:**

The biofilm assay showed 58.9% as high-grade and 31.5% as low-grade biofilm formers, while 9.58% were non-biofilm formers. Molecular characterization of the *csgA* gene showed 20.54% (15/73) positivity. The strains that were imipenem resistant also showed the *csgA* gene to be present (100%; 15/15), with 60% (9/15) and 20% (3/15) for meropenem and doripenem resistance respectively. A crystal violet assay for determining cell viability was done in vitro, which gave Minimum biofilm inhibition concentrations of 50% (MBEC50) at 25 µl and 90% (MBEC90) at 50 µl. The docking analysis done *in silico *showed benzofuran to possess the lowest binding energy and highest hydrogen bond interactions.

**Conclusion:**

The results indicate benzofuran, from the *O. sanctum* essential oils, to be effective in targeting the *csgA* gene among CRAB strains. Additionally, validation of these findings through *in vivo* studies is required.

## Introduction

*Acinetobacter baumannii*, *a* Gram-negative coccobacillus, is currently a major nosocomial pathogen (1) and its recent emergence (2), has marked it as one of the six most dangerous nosocomial pathogens by the World Health Organization (WHO) (3). It is a common inhabitant of soil, thus mistaken often as a soil pathogen (4). However, it is frequently isolated from hospital environments (5). Pneumonia, bacteremia, urinary tract infections, and meningitis are some commonly caused diseases by this opportunistic pathogen, among the immuno-compromised (6). The pathogen is also associated with clinical infections among patients in intensive care units (7). The pathogen’s inherent resistance mechanisms and biofilm-forming capability make it multi-drug resistant (8). The exorbitant mortality rates in such diseases, is therefore, due to the multi-drug resistance of the pathogen, making it a herculean task to devise treatment strategies and control the progression of the diseases (9). 

The *csgA* gene operon is exclusively associated with biofilm formation (10) facilitated by curli fibers (11), attributing to the organism’s virulence (12). The curli fibers expressed by the c*sgA* gene allow adhesion and invasion of *A. baumannii* to the epithelial cells of the host through extracellular matrix proteins (13). These fibers interact with the proteins and elicit an immune response in the host which further permit the organism to disseminate deeper into the tissues (14). Besides, they also slow down the activity of clotting factors (15), causing sepsis in the blood. This is noted by demonstration of *csgA* antibodies in the serum of sepsis patients (16). 

Genomic and proteomic diversity of the *csgA gene* operon, that regulates curli expression has been analyzed in detail amidst Eubacteria (17). The presence of two subunits viz., *csgA* and *csgB *has been documented accordingly. Among these, the c*sgA* subunit is the major subunit that assembles itself to form the cross-beta structures of the curli fibers (18). Meanwhile, the *csgB* subunit, being the minor subunit, is responsible for specific nucleation of the *csgA* associated fibers in biofilms (19). The *csgA* mediated curli fibrils are resistant to chemical and proteolytic degradation, enabling *A. baumannii* to thrive in harsh environmental conditions. Co-occurrence of *A. baumannii* showing carbapenem resistance and the *csgA* gene has also been reported in earlier studies (20). 

Hence, targeting the *csgA *gene might be an alternate method to confront drug-resistant strains of *A. baumannii*. Of the numerous natural herbs from India, *Ocimum sanctum* is believed to be the ‘Queen of Herbs’. Phytochemical analysis found saponins, tannins, alkaloids, glycosides, and several medically significant compounds were present (21). In a previous study (22), many bio-compounds extracted from *O. sanctum* have been reported for their anti-bacterial, anti-inflammatory, and anti-oxidant nature (23). Hence, in the current study, the *csgA *gene present in multi-drug resistant *A. baumannii* was molecularly characterized, and the ligands estragole, eugenol, methyl eugenol, benzofuran, naphthalene, and citral from *O. sanctum, *were docked *in silico*, against the *csgA *gene. 

## Materials and Methods

***Detection of biofilm formation using semi-quantitative adherence assay***

Cells cultured in a flat-bottomed, microtiter plate with 96 wells, were assessed for biofilms produced by specifically drug-resistant strains, as done in a previous study (24). For each strain, the assay was done thrice, in trypticase soy broth (HiMedia, Mumbai, India) using 200 µl of the fresh broth culture, containing 0.25% of glucose (w/v). After incubating it with a negative control (broth + 0.25% glucose) and a positive control (an earlier detected *A. baumannii* strain that can form biofilm), for 24 hr at 37 °C, phosphate-buffered saline (PBS) was used to wash the plates for removing the free cells. Fixing adhered bacteria with 95% ethanol for 5 min was followed by drying the plates. Lastly, 100 µl of 1% w/v crystal violet solution (HiMedia) was used for staining the wells, with excess stains removed after a resting period of 5 mins using distilled water. The wells were dried and the measurement of optical density (OD) with a plate reader, at 570 nm (OD_570_) wavelength was done. The results of biofilm formation were graded accordingly into high (OD_570_ greater than or equal to 1), low (0.1 less than or equal to OD_570_ which is lesser than 1), or negative (OD_570_ less than 0.1) values (25).

***Genomic DNA Extraction***

*A total of 73 strains of A. baumannii showing multi-drug resistance *as used in our previous studies (26, 27) *maintained at -*80 °C in 80%/20% (v/v) glycerol from our repertoire, were retrieved in LB medium. All strains were cultured in Mac Conkey agar with incubation at 37 °C for 24 hr. The Qiagen DNA extraction kit was used for genomic DNA extraction, which was done according to the instructions of the manufacturer, following which it was stored at 20 °C for future use.

***Detection of the csgA gene by PCR and sequencing***

Detection of the *csgA* gene by PCR was achieved using primers (28) the PCR conditions are enlisted in Table 1. Genomic DNA was amplified using a programmable thermal cycler [Eppendorf Mastercycler, Germany]. Using ethidium bromide containing 1.5% agar gel, 15 µl of the PCR product was prepared at 90V in a Tris borate buffer for 40 min alongside an appropriate 1KB DNA ladder marker. The *csgA* amplicon products were bi-directionally sequenced from forward primers and reverse primers, using a BigDye Terminator Cycle Sequencing Kit, a Bio-edit sequence analyser, and a 3730XL Genetic analyser. Lastly, the sequences were subjected to a BLAST analysis for similarity search of the nucleotides and were aligned by default parameters for Multiple Sequence Alignment by the ClustalW software.

***Inhibitory effect of O. sanctum essential oils***

*Source of plant extract*

Aerial parts of freshly cultivated *O. sanctum* plants were harvested, and essential oils were obtained by the hydro-distillation method. The extracted oil was dried to remove the excess water by adding anhydrous sodium sulfate. Following this, it was stored in dark vials at 4 °C. 

*Antibiofilm assay*

A flat-bottomed, polystyrene microplate with 96 wells was used to assess the effect of *O. sanctum* essential oils on *A. baumannii* mediated biofilm formation as described earlier (29). In brief, *A. baumannii* that were *csgA* positive were prepared into suspensions, in sterile trypticase soy broth, and the 0.5 McFarland standard suspension was used. Control wells for comparison, with medium, organism, and oil suspensions were included. After incubating the plates for 24 hr at 37 °C, the supernatant was discarded and sterile distilled water was used to remove the free-floating cells. They were kept undisturbed for 30 min to allow air-drying. Once the wells were dry, an aqueous solution of 0.1% crystal violet was used for staining them; they were let to take up the stain for 15 min. The plates were washed thrice to remove the stains, with distilled water. As a final step, the wells were solubilized by adding 250 µl of ethanol, and a plate reader at 570 nm was used to measure the absorbance. The equation 1 – (Test_OD570_/Control_OD570_) / 100 gave the % of Inhibition. The concentrations showing 50% and 90% inhibition of the biofilm formed were determined as the minimum biofilm inhibition concentration (MBIC) (30). 

*csgA retrieval and optimization*

The crystal structure of the gene of interest was obtained from the RCSB Protein Data Bank (http://www.rcsb.org/pdb) and was optimized by adding hydrogen atoms. The atoms of the proteins were assigned electronic charges and Kollman United Atoms Force-fields, with the help of the AutoDock Tool –1.5.6. The *csgA* gene’s three-dimensional form was developed using the RasMol tool. 

*Preparation and optimization of ligands*

The Chemsketch software was used to visualize the structural configurations of the bio-active derivatives viz., estragole, eugenol, methyl eugenol, benzofuran, naphthalene, and citral from *O. sanctum*, which were drawn and generated as 3D structures. The ligands that were selected, were saved as MOL files, after which they were converted and saved in the PDB format, using the Open-label molecular converter program.

*Molinspiration assessment for drug-likeliness*

Molecular descriptors including logP for partition co-efficient, the compounds’ molecular weights, and the hydrogen bond acceptors’ and donors’ counts relating to their membrane permeability and bioavailability, were assessed by a Molinspiration assessment program (31). Further evaluations on absorption, distribution, metabolism, and elimination (ADME) exhibited by the ligands were assessed in lieu of the rule of five proposed by Lipinski (32). 

*Drug-ligand interactions by docking*

The affinity between estragole, eugenol, methyleugenol, benzofuran, naphthalene, and citral, with the *csgA *gene of *A. baumannii*, was interpreted by docking, using the AutoDock tool. Using an auxiliary Autogrid program, the *csgA* protein was embedded in preset grid maps, one assigned each for a type of the atoms present in the compound that is being docked. The parameters of 12–10 and 12–6 given by Lennard–Jones, were applied to model all H–bonds and the van der Waals forces, respectively. The evaluation of the force field encompassed two steps viz., the intramolecular energetics from unbound states and bound conformations, and was given by the equation ∆G = ∆G_vdw_ + ∆G_hbond_ + ∆G_elec_ +∆G_tor_+ ∆G_desolv_ (33). 

*Visualization of docking*

The Discovery Studio Visualizer was used to visualize hydrogen bonds between estragole, eugenol, methyl eugenol, benzofuran, naphthalene, and citral, with the *csgA *gene of *A. baumannii.* Their molecular dynamics, affinity for binding, and energy simulation, besides further docking assessments, were used as parameters to assess their relative stabilities. 

***Statistical analysis***

*The obtained results were analyzed for statistical significance using SPSS version 21.0 (SPSS Inc., Chicago, IL, USA). Fisher’s exact 2-tailed test and Chi-square assessment were applied at P-value < 0.05. The frequency of the csgA gene among the CRAB strains was assessed using Pearson’s correlation test.*

## Results

***Correlating the csgA gene with CRAB strains***

**Figure 1:**
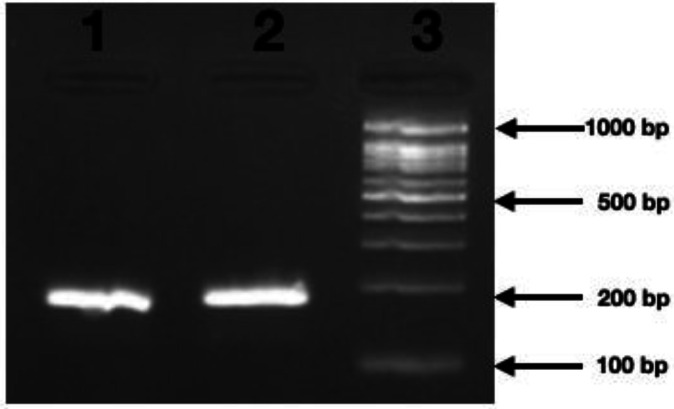
Electropherogram of *csgA *gene product of size 200 bp in lanes 1 and 2 with 1.5Kbp marker lane (M)

The biofilm assay among biofilm formers, indicated 43 strains (58.9%) under high grade, and 31.5% (23/73) under low grade, while 9.58% (7/73) did not form biofilm. Amidst the 43 strains showing high-grade biofilm-forming capability, all were imipenem resistant (100%; 43/43) followed by 76.7% (33/43) and 48.8% (21/43) for meropenem and doripenem resistance, respectively. Of the low-grade biofilm formers, all strains were resistant to imipenem, doripenem, and meropenem. The Pearson co-relation analysis yielded positive values suggesting the *csgA *gene to be occurring with CRAB strains (*P-value* <0.05). The molecular characterization of the gene, from the 73 genomes of CRAB strains, showed 20.54% (15/73) positive amplicons for the same (Figure 1). All strains resistant to imipenem showed the *csgA* gene to be present (100%; 15/15), followed subsequently by 60% (9/15) and 20% (3/15) resistant isolates against meropenem and doripenem, respectively. 

***Antibiofilm assay results***

**Figure 2:**
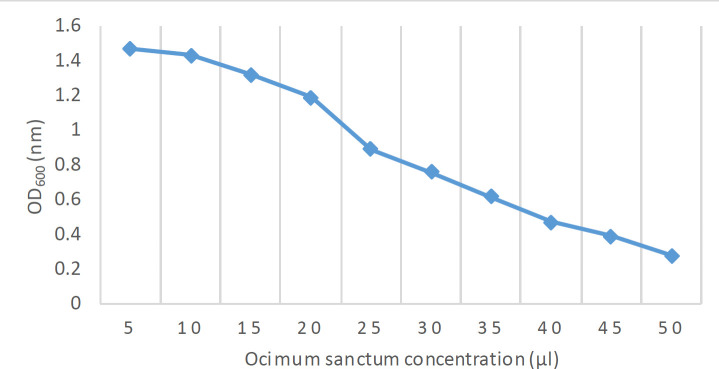
Graph showing the MBEC50 and MBEC90 values (OD_600 _nm) of the crude *Ocimum sanctum* extracts against biofilm-forming *Acinetobacter baumannii*

The crystal violet assay for cell viability showed MBEC_50 _at 25 µl, indicating that *O. sanctum* essential oils showed 50% inhibition of the biofilms against *csgA *positive strains (*P*<0.05). Likewise, MBEC_90 _was recorded at 50 µl, showing 90% inhibition (Figure 2). 

***csgA Protein structure retrieval***

**Figure 3:**
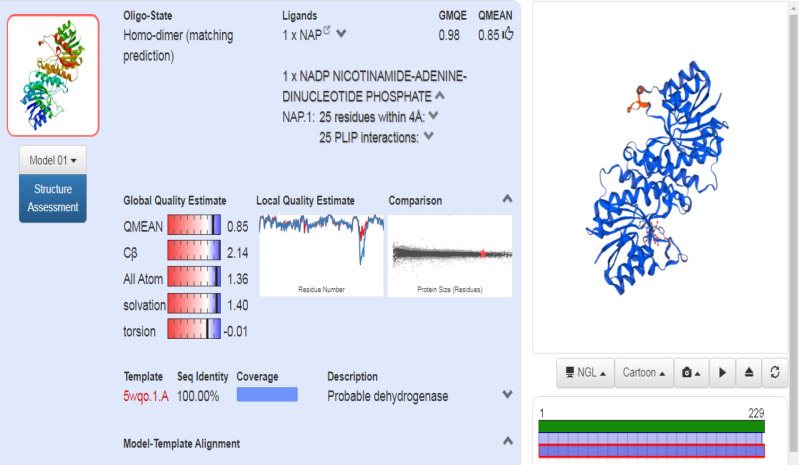
csgA structure prediction by homology modeling using the Swiss-Model web server

**Figure 4:**
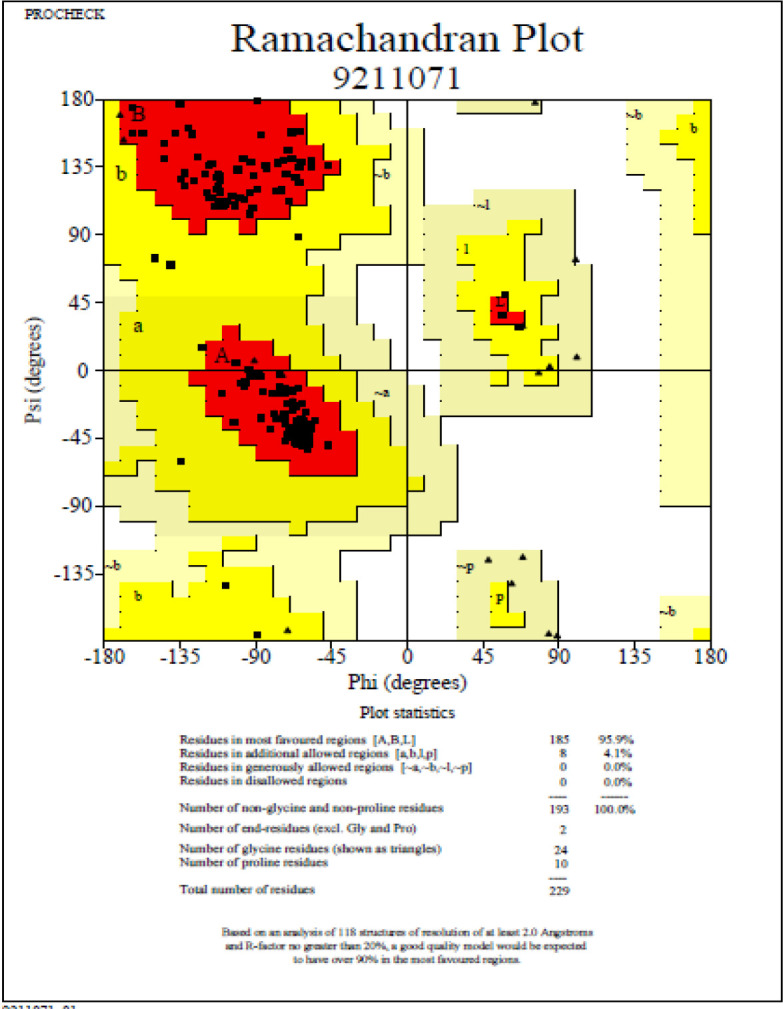
Ramachandran plot for validation of the predicted structure using SAVES Server – PROCHECK

**Figure 5:**
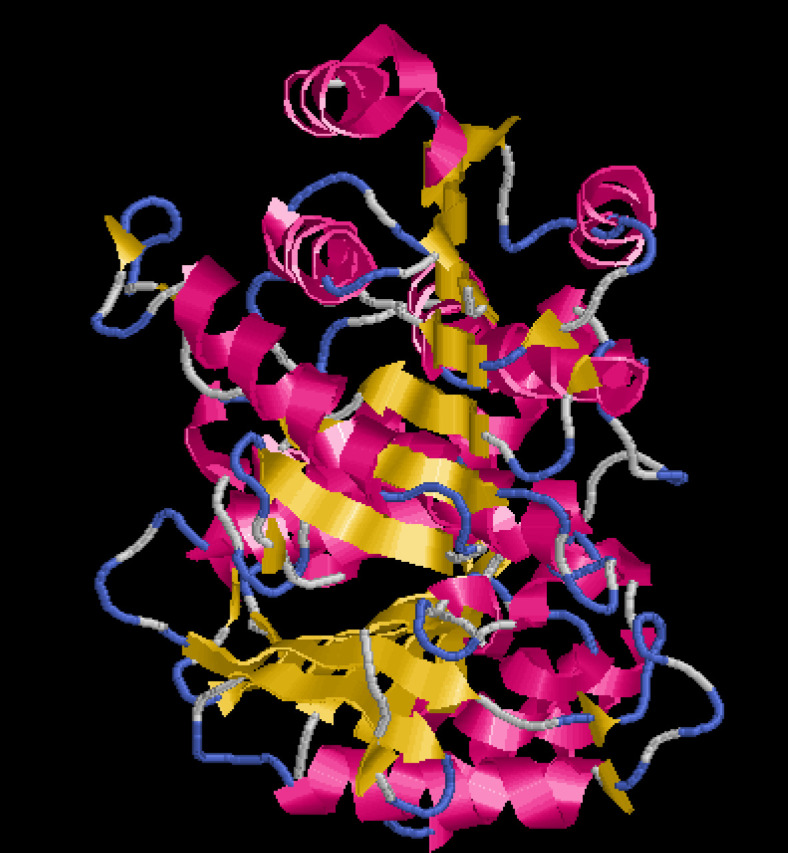
RasMol 3D structure of the csgA protein

The FASTA sequence for the *csgA *gene present in *A. baumannii *was retrieved from the Uniprot database (sequence ID A0A335NTF8)*.* Using the template 5WQO – A Chain, the structure of the *csgA* gene was modeled in the Swiss model server (Figure 3). The model showed 100% sequence identity compared with the template. Moreover, the Ramachandran plot indicated 95.9% of the residues to be in favored regions, with none in disallowed regions (Figure 4). The three-dimensional structure of the gene of interest was derived using RasMol in which the pink shade stands for alpha-helix, the yellow arrow denotes the beta sheets and the white shade denotes each turn (Figure 5).

***Retrieving the ligand– O. sanctum essential oils structures***

Optimization of the ligands was done using ACD Chemsketch software and the Open Babel Molecular Converter tool was used to convert them into a suitable format. Their two and three-dimensional structures and their SMILES format are shown in Figures 7 and 8.

***Drug likeliness parameter assessments***

Predictions of the bioactivity of estragole, eugenol, methyl eugenol, benzofuran, naphthalene, and citral, with the *csgA *gene of *A. baumannii*, were inferred from the findings of the set default parameters with predicted scores and tabulated in Table 2. Benzofuran was observed to be the most capable candidate as a drug. Following it was eugenol, as the second most capable of targeting the *csgA *gene.

***Docking analysis of O. sanctum derivatives against the csgA gene of A. baumannii***

Based on the ligand-receptor structures amongst those that were docked, as well as the lowest energy, and the minimal solvent accessibility, the most suitable conformers were chosen with the aid of the Lamarckian Genetic Algorithm (LGA). The ball and stick models of hydrogen bond interactions in estragole, eugenol, methyl eugenol, naphthalene, benzofuran, citral and the control, ceftazidime, against the *csgA *gene of* A. baumannii* were visualized using Acceryls Discovery Studio. These are given in Figure 4. The number of hydrogen bonds formed in concert with the torsional energy and the scores after the docking between the drug and ligands are also given in Table 3.

***Overall docking energies and interactions***

The binding energy together with other specific energies formed upon the interactions are shown in Table 4, with the energies reported in kcal/mol. The relative affinities of binding and the structure inhibitory activities for the *csgA* gene with estragole, eugenol, methyl eugenol, benzofuran, naphthalene, and citral present in *O. sanctum* essential oils was assessed using a computational algorithm for docking. Among the compounds, benzofuran showed the lowest free binding energy of –9.27 Kcal/mol with 6 hydrogen bonds, followed by eugenol with a free binding energy of –5.16 Kcal/mol and 4 hydrogen bonds. Citral and methyl eugenol showed three and two hydrogen bond interactions, respectively, with the binding energies of –4.87 Kcal/mol and –5.02 Kcal/mol, each. The remaining two compounds, estragole and naphthalene, showed free binding energies of –7.6 Kcal/mol and –4.71 Kcal/mol, respectively, with 1 hydrogen bond. Other interactions formed from the interactions are also recorded under Table 5. Hence, the bio-compounds in the essential oils of *O. sanctum* showed a good binding affinity with the *csgA *gene. The compound benzofuran was observed to be the best candidate, when compared with the others, to target the *csgA* gene, showing the best docking scores with 12 and 4 van der Waals interactions and alkyl/π-alkyl interactions, respectively.

## Discussion

Biofilm formation in *A. baumannii* is exhibited as a progressive process, involving the adhesion of bacteria to a surface, micro-colony development, followed by biofilm formation and maturing, and detachment leading to further colonization (34). Amidst various biofilm-associated genes, the *csgA *gene-mediated curli fibrils (35) are specifically known to transform a cell from its planktonic or single-celled state to a colonized community or a biofilm system (36). This further attributes to the pathogenicity and virulence of the biofilm-forming *A. baumannii* bacteria (37). Therefore, the current investigation was undertaken for the molecular characterization of the *csgA* gene and highlight its correlation with *A. baumannii *strains showing multi-drug resistance. In addition to this, the study also aimed to throw light on how to curb the development of biofilms in *A. baumannii,* as an alternative strategy to combat the menace of its survival. To substantiate this, the study has incorporated assessments on the activity of essential oils from *O. sanctum* against biofilm formed by *A. baumannii* strains that are *csgA* positive. 

Previous studies have demonstrated the incidence of the *csgA* gene in *A. baumannii,* with 63% in a genotypic detection in associated with the biofilm formation-based virulence (38). In view of this, the occurrence of 20.54% positive amplicons among the CRAB strains observed in this study hypothesize the role of the *csgA* gene in enhancing the resistance and virulence of the *A. baumannii *strains showing multi-drug resistance. In contrast to this, previous literature had documented the absence of the gene in multi-drug resistant strains, highlighting the role of other genes in biofilm development (39). It is inferred that the role of the biofilm produced by the *csgA* gene, might differ as it is expressed (40) and thus, a periodical screening would give insights regarding the gene’s potential role in virulence (41). 

The gold standard crystal violet staining, to assess the activity of the chosen essential oil compounds against biofilm, was employed for its cost-effectiveness and ability to give rapid and adaptable laboratory results (42). Essential oils of *Ocimum *sp., have already shown to exhibit a good antibiofilm activity, as recorded in previous literature (43,44), against drug-resistant strains of *Staphylococcus aureus* (45) and *Escherichia coli *(46)*. *No study however, has vividly documented the same against MDR (multi-drug resistant) strains of the organism of interest in the current study. Hence, the present study throws light on the antibiofilm activity of *O. sanctum* essential oils with a high inhibitory effect on the formation of biofilm in *csgA* positive *A. baumannii *strains. 

The present study also intended to target the *csgA* gene-mediated biofilm, using natural bioactive compounds, for which essential oil compounds from *O. sanctum *were selected. The herb *O. sanctum* is easily available in India and several of its phenolic compounds have been structurally characterized in detail (47). Moreover, various bio-activities of the plant have been evaluated and reported earlier (48). It has also been observed that essential oils from *O. sanctum*, encompass potent hydrophobic bio-compounds which are highly suitable for nano-formulations (49). According to previous literature, essential oils from the extracts of *Tulsi* possess a promising antibacterial property (50), attributed to the presence of 71% eugenol, in their compositions (51). In view of this, *O. sanctum* and its potent bio-compounds were selected for the drug–ligand interactions. 

Since characterization of essential oils from *O. sanctum *has already been extensively analyzed, this study concentrated on *in silico* evaluation of the six bioactive compounds chosen as per findings in previous literature (52). In accordance with this, csgA was efficiently targeted by an *in silico* docking analysis using computational bio-informatic tools and databases. Based on factors like pose and strength core (53), a suitable ligand–receptor complex was obtained. The Biovia system was used to identify the number of hydrogen bonds and the bonding energies to obtain the best fit, with a high score, for benzofuran. In comparison with the control ceftazidime, albeit of its best binding scores, the strains selected for the study were resistant to the drug and many studies do document the same. Thus, the present investigation suggests benzofuran as the best candidate of choice for an alternative therapeutic strategy against drug-resistant strains of *A. baumannii.* Benzofuran is considered as a potent bio-compound from *O. sanctum* with a minimum inhibitory concentration (MIC) value of 29.76–31.96 lmol/L as observed in an earlier study, indicating a vital anti-microbial activity (54).

The Molinspiration assessments, based on the specific parameters, showed high drug likeliness of the chosen compounds from *O. sanctum,* against *A. baumannii* possessing the *csgA *gene. The topological polar surface area (TPSA), in view of the drug absorption and bio-availability, indicates smooth and efficient binding of the selected ligands to the *csgA* protein. It is known that a TPSA value, equal to 140 Å or higher, indicates less absorption and oral bioavailability of the drugs (55). However, for the selected compounds from *O. sanctum *in the present study, TPSA values were less than 140 Å. Thus, the possibility of these compounds being formulated as drugs is highlighted from this finding. 

Docking analysis involved investigating the free binding energy (∆G) to predict the ligand binding with the csgA. The LGA assessed the binding conformational landscape of estragole, eugenol, methyl eugenol, benzofuran, naphthalene, and citral with the *csgA *gene. The docking scores of the csgA with the selected ligands showed a prominent relationship between the energies of the affinity of binding, stability, and low docking scores. Accordingly, the inter-molecular energies, van der Waal’s forces, and torsional energies were comparatively higher for benzofuran followed by eugenol. Thus, it has been theoretically demonstrated that benzofuran from *O. sanctum* exhibited the highest inhibitory activity against the *csgA *gene-mediated formation of biofilm among MDR *A. baumannii* strains. 

**Table 1:** Primer sequence and PCR conditions to detect *csgA* gene in MDR of *Acinetobacter baumannii* strains

**Gene of target**	**Primer details**	**Annealing temp**	**Amplicon size**
csgA	ATTTACCAGGATGGGCCGTG GCGCCACAACCAAGCAATTA	55	200 bp

**Table 2:** Molinspiration assessments of *Ocimum sanctum* ligands

**Bio-compounds**	**Mol. wt**	**H-Donor**	**H-Acceptor**	**miLogP**	**Rotatable bonds**	**nViolations**	**TPSA (Ǻ)**	**Volume**	**N atoms**
Estragole	148.21	0	1	2.82	3	0	9.23	154.12	11
Eugenol	164.20	1	2	2.10	3	0	29.46	162.14	12
Methyleugenol	18.47	0	2	2.41	0	0	18.47	179.67	13
Benzofuran	374.35	0	9	4.49	6	0	119.35	318.05	27
Naphthalene	220.36	0	1	4.66	1	0	17.07	238.11	16
Citral	152.24	0	1	3.65	4	0	17.07	169.74	11
Ceftazidime	546.59	4	13	–5.68	9	2	191.23	439.78	37

**Table 3:** Interactions of ligands derived from *Ocimum sanctum* essential oils with csgA protein

**S. No**	**Bio-active compound**	**csgA**		**Atom in bio-active compound**	**Distance (Ǻ)**	**Docking energy (Kcal/Mol)**
		**Residue**	**Atom**			
1.	Estragole	VAL184	N	O	2.92	–4.71
2.	Eugenol	TYR151 SER135 SER135 SER135	OH OG OG OG	O O O H	3.04 2.82 2.50 1.75	–5.16



3.	Methyleugenol	LEU59 LEU59	N N	O O	3.01 3.05	–5.02

4.	Benzofuran	ARG33 LYS155 ASN83 GLY14 ILE13 ILE13	NH2 NZ ND2 N N N	O O O O O O	3.12 2.66 2.83 2.97 3.07 2.85	–9.27





5.	Naphthalene	VAL184	N	O	2.75	–7.6
6.	Citral	ASN83 GLY14 ILE13	ND2 N N	O O O	2.89 2.93 2.78	–4.87



7.	Ceftazidime	ARG33 ARG33 ARG11 TYR151 LYS155 GLY85 GLY85	NH2 NE NH2 OH NZ O N	O O O O O H O	2.97 2.92 3.06 3.07 2.88 2.03 2.78	–9.94


**Table 4:** Interaction scores of *Ocimum sanctum* against csgA of *Acinetobacter baumannii*

**Compounds**	**Number of h-bonds**	**Binding energy**	**Ligand efficiency**	**Intermolecular energy**	**vdW + Hbond + desolv energy**	**Electrostatic energy**	**Torsional energy**	**Total internal Unbound**
Estragole	1	–4.71	–0.43	–5.61	–5.57	–0.04	0.89	–0.21
Eugenol	4	–5.16	–0.43	–6.36	–6.25	–0.11	1.19	–0.73
Methyleugenol	2	–5.02	–0.39	–6.21	–6.24	0.03	1.19	–0.36
Benzofuran	6	–9.27	–0.34	–11.06	–9.53	–1.54	1.79	–0.77
Naphthalene	1	–7.6	–0.48	–7.9	–7.83	–0.07	0.3	–0.29
Citral	3	–4.87	–0.44	–6.06	–5.99	–0.07	1.19	–0.27
Ceftazidime	7	–9.94	–0.27	–13.22	–10.81	–2.41	3.28	–2.35	

**Table 5:** *Ocimum sanctum* bio-compounds overall interactions with csgA

**Bio-compounds**	**H-bonds interactions**	**vW interactions**	**π-σ /** **π-π / amide-π stacked interactions**	**alkyl/π-alkyl interactions**
Estragole	1	8	-	2
Eugenol	4	10	-	3
Methyleugenol	2	9	1	2
Benzofuran	6	12	-	4
Naphthalene	1	11	-	10
Citral	3	5	-	5
Ceftazidime	7	14	1	5

**Figure 6:**
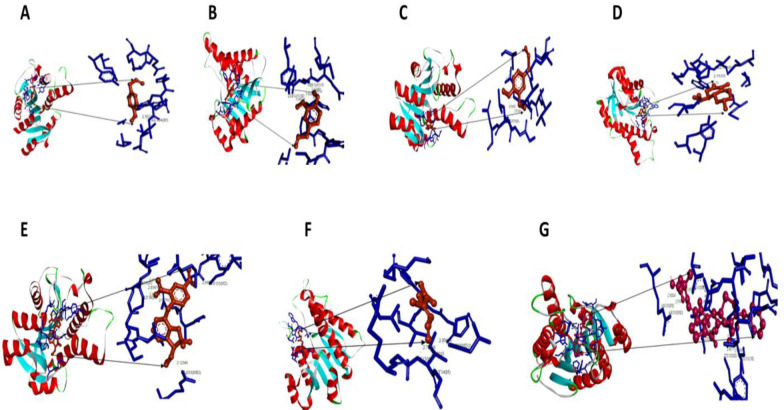
Docking visualization of csgA gene with A – Estragole, B – Eugenol, C – Methyleugenol, D – Benzofuran, E – Naphthalene, F – Citral, G – Ceftazidime

## Conclusion

The tenacious pathogen, *A. baumannii, *can be inhibited by targeting the biofilm mediating *csgA *gene. This further, can be effectuated by derivates of the essential oils of a commonly available herb, *O. sanctum*. Among the six bio-compounds chosen for the study, benzofuran and eugenol have shown favorable results in lieu of the study. Their inhibitory effect on the *csgA* gene has been substantiated *in vitro*, by the results of the antibiofilm assay, docking analysis, and Molinspiration assessment. Suitable TPSA values have also indicated that they can be considered for drug development. Therefore, the study has thrown light on an alternative means to address the menace of the recently progressing nosocomial pathogen, the MDR strains of *A. baumannii*. 

## Fundind Source

Self-funded study.
